# Determinants of the little auk (*Alle alle*) breeding colony location and size in W and NW coast of Spitsbergen

**DOI:** 10.1371/journal.pone.0212668

**Published:** 2019-03-06

**Authors:** Liliana Katarzyna Keslinka, Katarzyna Wojczulanis-Jakubas, Dariusz Jakubas, Grzegorz Neubauer

**Affiliations:** 1 Department of Vertebrate Ecology and Zoology, Faculty of Biology, University of Gdańsk, Gdańsk, Poland; 2 Laboratory of Forest Biology, Wrocław University, Wrocław, Poland; University of California Santa Cruz, UNITED STATES

## Abstract

Many seabirds breed in large aggregations, making it difficult to estimate their population size and habitat preferences. This knowledge is particularly important considering their function in food webs and ecosystem services. In this study, we investigated the factors affecting distribution and abundance of the little auk *Alle alle*, a seabird considered a keystone species of the Arctic ecosystem. We performed the study on the W and the NW coast of Spitsbergen. Using Generalized Additive Models (GAMs) and Conditional Inference Tree (CIT) we examined factors related to presence/absence and size (estimated number of breeding pairs) of the little auk colonies. We also tested the nesting preferences for geographical features such as aspect, slope angle, altitude, solar radiation, rock type, and distance to foraging grounds. Our findings indicate that the occurrence of little auk breeding colonies is non-random and highly attributed to environmental factors. The probability of colony occurrence was significantly associated with altitude (negative relationship; preference to sites situated lower), solar radiation (positive relationship; the higher radiation, the more likely colony occurrence) and slope (positive relationship; the steeper a slope, the more likely colony occurrence), whilst aspect appeared non-significant (though the probability of colony occurrence peaked at southern slopes). Colony size was significantly associated with rock type (larger colonies in amphibolite and quartzite). The distance to foraging grounds did not appear to affect the probability of colony occurrence and size, implying that birds may choose optimal breeding sites at the cost of longer foraging flights. We estimated the Spitsbergen little auk breeding population at 728 529 (5–95% CI: 479 312–986 352). Spitsbergen comprises *ca* 1.9% (95% CI: 1.2%–2.7%) of the world breeding population and represents the third most important breeding area for the species, following the W and the E coast of Greenland.

## Introduction

Nest site selection is a key component of successful breeding in seabirds [1]. Optimal conditions at a breeding site are shaped by multiple factors, including both abiotic and biotic components as well as their interactions [2], operating at various scales. At macro- and meso-scales birds may search for a site with appropriate climate characteristics (e.g. time sufficient to complete the breeding season), nest site topography (e.g. appropriate site for nest building/anchoring), and biotic conditions (e.g. location of foraging areas at a cost-effective distance from the nesting site, low predation pressure). At a micro-scale birds may search for more specific climate and topographic conditions that influence nest site availability at a given time, such as nest temperature and wind exposure.

Colonial seabirds often nest in coastal habitats, where, once established, colonies remain occupied in a given location for a long period of time. Seabirds typically exhibit a high site fidelity and philopatry [3,4]. Even when conditions are temporarily unfavourable, (e.g. low food availability, high predation pressure etc.) seabirds often return to the same breeding location in the following seasons [5]. On the other hand, when unfavourable conditions persist for a longer time, demography of local populations can be affected. Colony size may then carry information about current environment conditions [6,7]. Recognizing habitat preferences of seabirds, and their population size are important steps in the process of conservation management.

**Fig 1:**
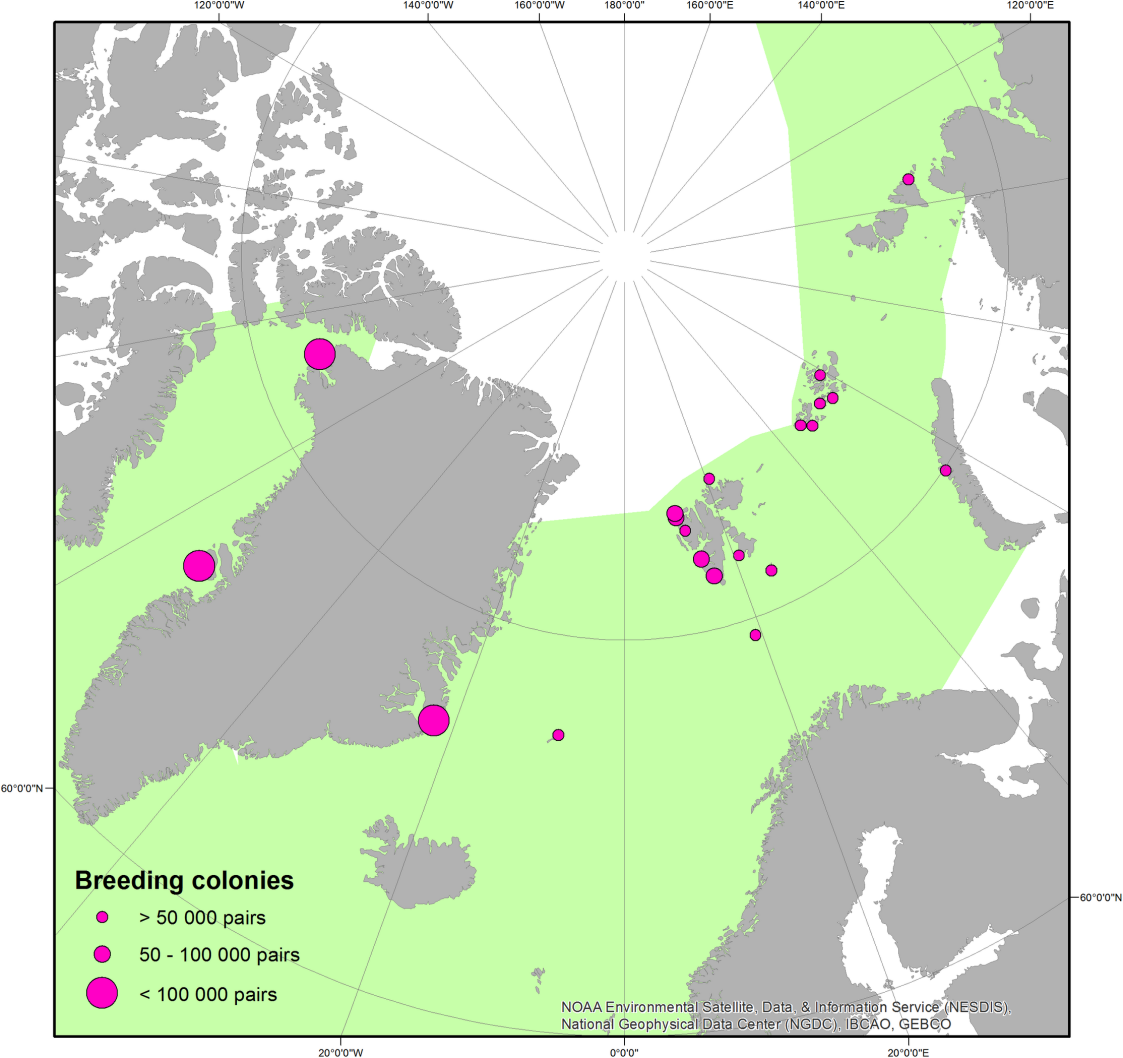
Distribution of foraging areas and breeding colonies of the little auk. Frame indicates the study area. Green colour represents the range at sea. Dot size corresponds to colony size (number of breeding pairs). Source of data summarized in Table 5. Background (bathymetry and contour map): NOAA Satellite Maps.

In this study, we focus on the little auk (or dovekie, *Alle alle*), a small planktivorous seabird breeding exclusively in the High Arctic (Fig 1). Due to its abundance (37–40 million pairs globally) [8], the little auk is an important component of the High Arctic ecosystem. By transporting large amounts of organic matter from sea to land, fertilizing the nutrient-deprived Arctic tundra [9,10], the little auk plays a role of an ecosystem engineer, transforming terrestrial ecosystems across the High Arctic [11]. The population of the little auk in the High Arctic may soon undergo changes due to the ongoing climate change [12]. In fact, modelling of the species’ future distribution under scenarios of 1°C and 2°C sea surface temperature increase predicts losses of suitable foraging habitat for the majority of colonies on Svalbard, and, as a result, declines in the local populations [12]. Considering the ornithogenic fertilization effect of little auk colonies, the birds’ retreat may have serious negative implications for marine and terrestrial Arctic ecosystems. Until now, the population size of the little auk and factors potentially affecting it are poorly recognized. Due to nesting in burrows, usually on steep coastal slopes, the little auk is a challenging species to accurately measure its population size. There are only few studies focused on the little auk colonies distribution and size (e.g. [13–16]). Consequently, current estimates are typically imprecise and incomplete, with habitat preferences having never been systematically examined.

The aim of our study was firstly to verify location and size of the little auk colonies on the W and NW coast of the Svalbard Archipelago (Norway). The second aim was to determine what factors are associated with colony presence/absence and size. For this purpose, we considered the following predefined factors: a) distance between the colony and main foraging grounds; b) altitude c) aspect of the slope d) angle of the slope, e) solar radiation, and f) rock type. We expected birds to nest at a cost-effective distance from the foraging grounds, with the largest colonies located closer to the main foraging grounds. Considering shadow effect of neighbouring mountains, affecting snow melting in the spring, birds should prefer southern aspects ensuring higher solar radiation and providing a faster release of nesting chambers. Considering slope stability, we expected little auks to breed on moderate slopes (20–30°), reducing risk of rolling boulders, but also avoiding flat topographies, which impede the species predator response behaviour. We expected that little auks would avoid nesting in sedimentary rock scree, built by small rocks, with fewer suitable nesting chambers and less stable slopes than locations covered with bigger rocks.

## Materials and methods

The study was conducted under permission of the Governor of Svalbard.

### Colony surveys

**Fig 2:**
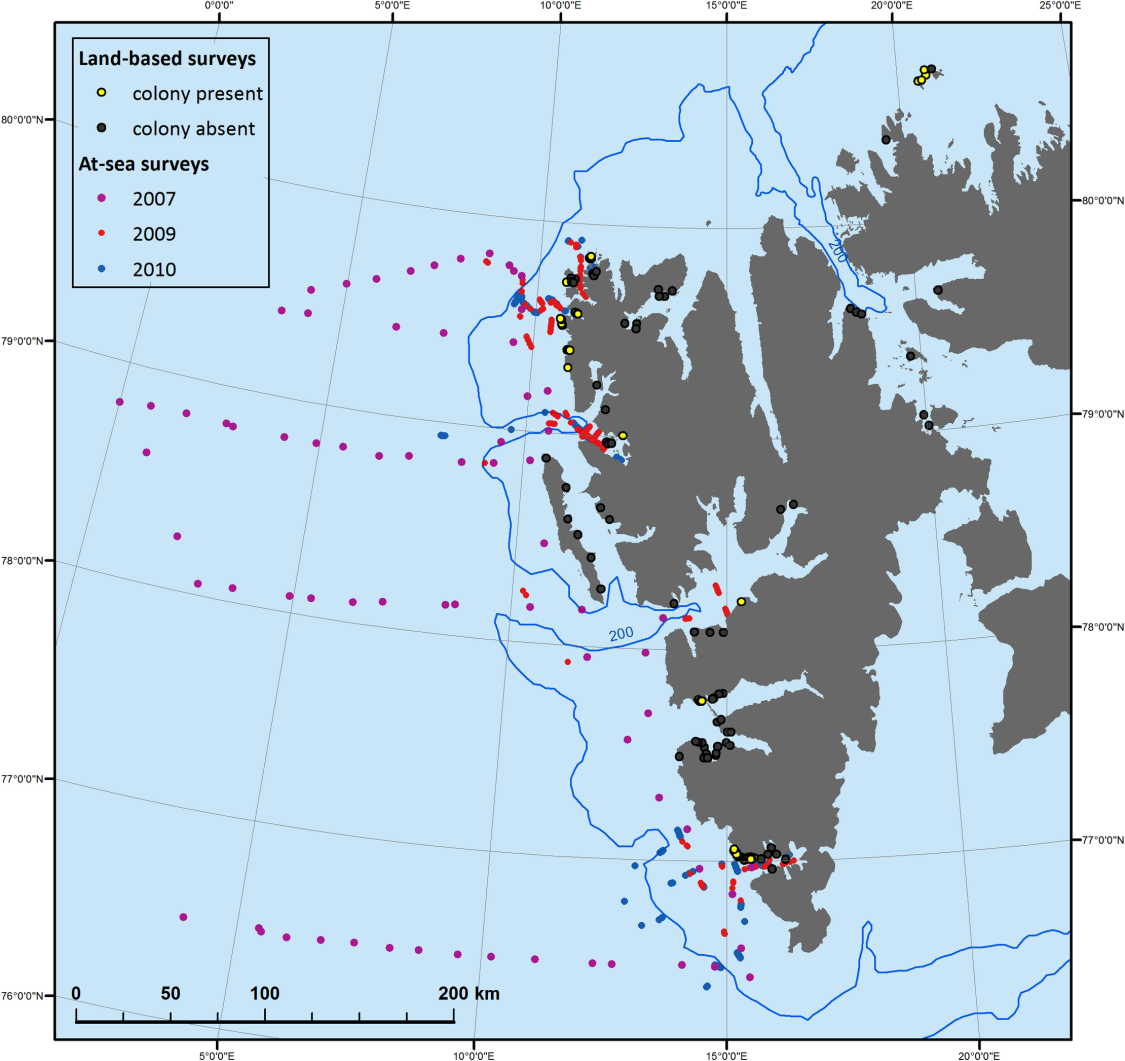
Study area with location of visited sites with (yellow dots) and without little auk colonies (black dots). Dots at sea indicate ship cruises during at-sea surveys of foraging birds. Background (bathymetry and contour map): NOAA Satellite Maps.

To establish location and size of little auk colonies, surveys were conducted along the N and the W coast of Spitsbergen (the biggest island in Svalbard Archipelago, Norway) during the breeding seasons between 2009 and 2015 (Table 1 and Fig 2). The choice of sites surveyed was based on known breeding locations (http://svalbardkartet.npolar.no/) and logistic constraints. At each visited site, being a potential little auk habitat [17], we carefully searched for evidence of bird presence (visible/audible adults and/or chicks, flocks of birds flying in circles above the scree, apparent faeces deposition etc.). Occupied colony patches were then examined to establish colony borders, based on bird presence. Patch (polygon) coordinates were marked along the border of the patch; the number of nodes depended on the complexity of local topography. A laser range-finder (TruPulse 360, USA) connected with a GPS receiver was used for setting coordinate points. Photographs (1–5 images) were taken in order to measure rock diameter within patches, using a 1 m levelling rod for a scale. This allowed for the later estimation of local nest site density. 143 colony patches were surveyed in total.

**Table 1:** A summary of surveyed little auk colonies and at-sea surveys performed during this study.

Year	Month	Area
*Colony surveys*		
2009	July	Hornsund (Ariekammen), Billefjorden (Petuniabukta)
2010	July	Hornsund (Revdalen, Gulliksenfjellet)
2011	July	Hornsund (Revdalen, Lechbotnen, Steinvikdalen, Treskellen, Burgerbukta, Gåshamna)
2012	June	Bellsund, Wedel Jarlsberg Land (Dunderdalen)
2013	July-August	Isfjorden (Bjørndalen), Prins Karls Forland, Kongsfjorden, Krossfjorden, Magdalenefjorden, Smeerenburgfjorden, Nordaustlandet (Depotodden), Sjuøyane
2015	June-July	Van Keulenfjord, Alkepynten, Blomstrandhalvøya, Krossfjorden, Danskøya, Amsterdamøya, Fuglefjorden, Liefdefjord, Andøyane, Hinlopenstretet, Nordaustlandet (Zeipelodden)
*At-sea surveys*		
2007	06.07–05.08	Greenland Sea (transect central point locations in Fig 2)
2009	24.07–06.08	
2010	22.07–03.08	

### Colony patch characteristics

For each colony patch, the following features were assessed: total area (in m2), number of nests and five environmental traits: elevation (in m a. s. l.), slope (in degrees, 0–90°), aspect (degrees, 0–360°), solar radiation in May (kWh), and geomorphology (rock types).

To establish the total area of patches, patch coordinates were transformed into polygons using ArcGIS 10.3 (ESRI, Redlands, USA). Rectified aerial images (Norwegian Polar Institute) of the studied area were used for cross-reference and checking the accuracy of a polygon positioning. The size of each colony patch was calculated using the “Calculate geometry” tool in ArcGIS 10.3. The size was corrected for the slope (measured in grades) using the mean angle of the slope of each colony patch and formula: *c* = *a* × cos(*α*) ^-1^, where *a* is the original (flat) area of the polygon, *α* is the mean angle of the slope of the polygon and *c* is the new, corrected area value of the colony patch [18].

To identify the number of nests in patches, every patch area was multiplied by the local nest density. The local nest density was based on a linear model relating mean rock size and nest densities known from the literature [13, 19, 20] and this study (rock diameters obtained from scaled photographs taken *in situ* (Table 2). An estimation of a nest density based on a stone size gives comparable results to other methods such as a video surveillance method [21]. The stone size-based estimation is less invasive than other nest searching methods which require long-lasting visits to colonies, and furthermore, direct nest counts are challenging because nests are typically situated deep below stones [21]. The estimates based on this method are not free form uncertainties, nonetheless it offers a higher degree of accuracy than knowledge available in that matter up to this day.

**Table 2:** Estimated density of nests (with 95% CrI in parentheses) and rock diameters in the surveyed little auk colonies. The density values marked as “this study” are the estimates of mean densities from the nest density model (see section “Colony patch characteristics” above and S1 Table).

Region	Average nest density [nest/m^2^]	Average rock diameter [m]	Rock diameter (RD) and nest density (ND) data source
Hornsund (Gulliksenfjellet)	1.99 (1.29–2.72)	0.61	RD, ND–this study
Hornsund (Fugleberget)	1.28 (0.90–1.68)	0.39	RD, ND–this study
N-W Spitsbergen	1.51 (1.03–1.99)	0.45	RD, ND—this study
Bellsund	1.44 (0.99–1.89)	0.43	RD, ND—this study
Isfjorden (Bjørndalen)	0.58 (0.21–0.97)	0.25	RD—literature (13)
Kongsfjorden	1.0	-	RD data not available, ND—averaged from Hornsund and Isfjorden nest densities
Sjuøyane	0.2	-	RD—data not available, ND—half of the Isfjorden nest density

We built a simple linear model: $\hat{D}$ = *a* + *b* × (log(*RS*)), where $\hat{D}$ is the nest density (number of nests / 1 m^2^), and *RS* is the mean rock size (in cm) per plot. It explained more variation (*R*^*2*^ = 0.70) than a straight linear model (*R*^*2*^ = 0.61) or any other relationship. This model had a curvilinear relationship between a rock size and a nest density and allowed to estimate nest densities (along with their uncertainty) across the range of observed rock sizes except for very small ones (smaller than 10 cm in diameter), which were, however, not recorded in the studied colonies. To make inference valid with our small sample size (7 data points), we carried out a Bayesian analysis and fitted this model using MCMCglmm library [22] in R 3.2 (R Core Team 2015). We ran two Markov chain Monte Carlo simulations, with standard settings (13,000 iterations, a burn-in of 3,000 and a thinning rate of 10, resulting in a posterior sample of 1,000 per chain) which were fully sufficient for this small data set. Convergence was perfect as assessed visually, and with Gelman-Rubin-Brooks statistics ($\hat{R}$, 1.003 for both coefficients) computed in the coda library ([23]; S1 Table and S1 Fig). We then calculated expected mean nest densities and their 95% credible intervals from the posterior samples (presented in Table 2 as nest densities obtained in this study).

In two cases (Kongsfjorden and Sjuøyane) exact data on the rock diameter was not available, therefore the average nest density in these cases was based on values from other colonies of similar rock structure and known rock diameter. For a single colony in Kongsfjorden we decided to conservatively apply density of 1 nest/m^2^ to avoid overestimation. This density equals averaged value of nest denisties from Hornsund and Isfjorden. The colonies at Sjuøyane have similar morphology (rock diameter) to the Isfjorden colonies, therefore we assumed that the nest density should not exceed 0.2 nest/m^2^, what is equal to the lowest nest density documented in Isfjorden by Isaksen and Bakken [13]. Additionally, since the colony on Amsterdamøya is placed on a vertical cliff, it was impossible to estimate the nest density using the same method as for the other colonies. Thus, the number of pairs for this colony was estimated based on observations of birds flying in the neighbourhood of the cliff. Based on literature considering colony attendance pattern of little auks during the time of the visit in the colony–mid chick rearing period [24], we assumed that we observed 50% of nesting adults (Table 3).

**Table 3:** The surveyed colonies, and predicted number of little auk pairs. CI–confidence interval.

Region	Area	No of colonies	Predicted no. of nests per m^2^	Predicted number of nests	5% CI	95% CI
Bellsund	Ingeborgfjellet	15	1.44	35 814	24 665	47 165
Hornsund	Ariekammen	19	1.99	18 064	11 669	24 704
	Fugleberget	2	1.28	76 80	5 370	10 039
	Hyttevika	9	1.99	370 530	239 350	506 715
	Lechbotnen	7	1.99	30 893	19 956	42 248
	Revdalen	6	1.99	29 615	19 130	40 500
	Rotjesfjellet	16	1.99	68 596	44 311	93 808
	Torbjørnsenfjellet	6	1.99	66 513	42 965	90 960
Isfjorden	Bjørndalen	1	0.58	135	49	227
Kongsfjorden	Kongsfjorden	1	1.00	3 890	3 890	3 890
N-W Spitsbergen	Amsterdamøya	1	—*	500	500	500
	Fuglesangen	19	1.51	28 710	19 658	37 906
	Hamburgbukuta	11	1.51	36 515	25 002	48 210
	Magdalenefjorden	18	1.51	18 089	12 386	23 883
	Nilsenfjellet	7	1.51	8 164	5 590	10 778
Sjuøyane	Sjuøyane	5	0.20	4 821	4 821	4 821
			Total	**728 529**	**479 312**	**986 352**

* little auks nest there on vertical cliffs; the number of pairs was estimated based on bird counts close to the cliffs

Environmental traits were established in ArcGIS 10.3 using the Spatial Analyst toolbox based on Digital Elevation Models (DEMs, Norwegian Polar Institute, University of Silesia) in 20 m resolution. Solar radiation (Solar Radiation Tool) was calculcated separately for each month between May and August (little auk presence in colonies), and expressed as a sum of the radiation for these four months. Slope angle was calculated with the Slope Tool, aspect with the Aspect Tool and elevation was retrieved directly from the DEMs. Geomorphology data (type of rocks) were obtained from a geological map by the Norwegian Polar Institute [25].

### Main foraging areas of little auks

To identify foraging hotspots, at-sea surveys of foraging little auks were undertaken during the chick-rearing period in 2007, 2009 and 2010. The surveys were performed on board of s/y ‘Oceania’ research vessel (Institute of Oceanology, Polish Academy of Sciences) along the West coast of Spitsbergen in the shelf and off-shelf region of the Greenland Sea (77°–79° N, 0°-16° E). In total, 615 survey transects of different lengths (weather conditions and sail program dependent) were performed to cover potential foraging areas of birds from the examined colonies (Fig 2 and Table 1).

Birds were counted while sailing during calm to moderate sea conditions (0–4 force Beaufort scale according to World Meteorological Organization). Counts were performed from the vessel’s bridge within a range of 300 meters (i.e. from the bow of the ship to 90 degrees on the port and starboard side of the ship). All birds spotted in the established range were counted (both sitting on the water and flying).

**Fig 3:**
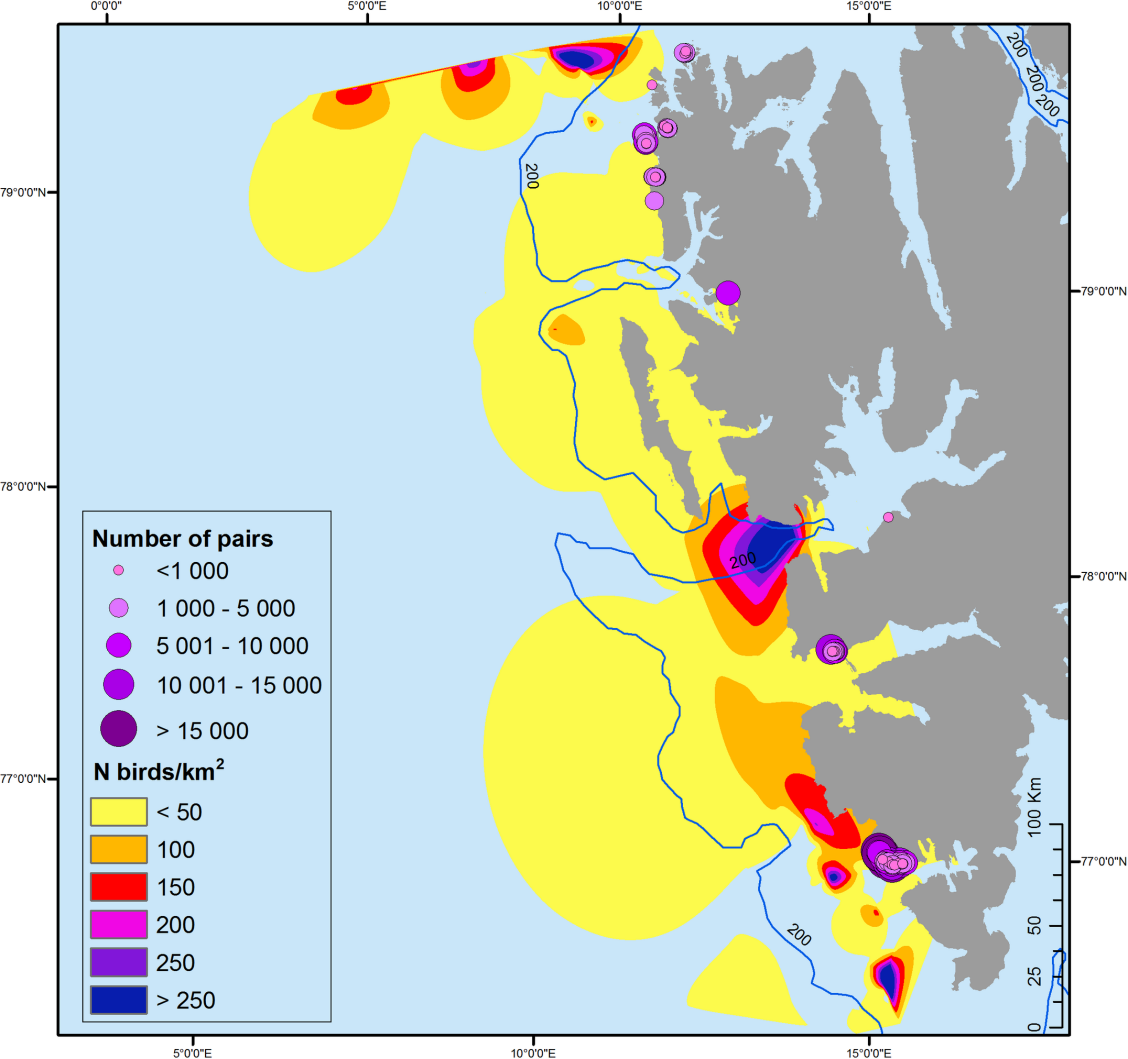
Location of the little auk foraging hotspots interpolated from data from at-sea surveys. Circles on land represent little auks colonies. The size of the circle refers to the estimated colony size (the number of breeding pairs). Background (bathymetry and contour map): NOAA Satellite Maps.

To identify the foraging hotspots, data from the three seasons of at-sea surveys was combined. The number of birds observed per 1 km^2^ was calculated by dividing the number of little auks observed along a transect by the transect area in km^2^ (transect length × 300 m). The foraging hotspots were established using the Natural Neighbour Interpolation tool in ArcGIS 10.3, with the hotspot being considered as the highest local concentrations of the birds at sea (the highest numbers of little auks observed per km^2^). The distance from the center of the colony to the foraging hotspots was calculated in ArcGIS 10.3 (Fig 3).

## Data analysis

### Factors associated with colony presence/absence

To investigate factors associated with colony presence/absence, we used true colony patches (n = 143) and randomly generated absence data (sites without colony patches) in ArcGIS software using the Create Random Points tool (n = 194 polygons). The absence locations were placed within 7 km from the coastline in order to cover all available potentially suitable nesting terrain. The interior (more than 7 km inland on average) was not taken into account because it is mostly covered by glaciers and ice sheets and there are no records of nesting little auks that far inland. The absence locations were placed also within 55 km distance from the foraging hotspots (twice the mean distance from the colony patch to the nearest foraging hotspot in the main concentration of little auks on the West coast of Spitsbergen: Hornsund). The size of all random polygons was the same (2 900 m^2^) and was set to the mean size of the measured colony patches. The random polygons were then validated visually based on the aerial images. The polygons located on glaciers, flat rocky islets (unlikely location of little auk colonies) or at known colony patches position were excluded leaving 194 polygons meeting all the above crtieria.

For the purpose of occurrence probability modelling, mean values of environmental traits calculated from the DEM raster cells (20 m size) falling into polygons were used to produce the colony-level (polygon-level) variables. All spatial traits were ln(x+1) transformed.

To explain a variation in colony presence/absence, generalized linear additive models (GAMs) with binary response and logit link function at the level of a ‘breeding site’ (true colony or unoccupied random polygon) were used [26]. With smoothing functions, GAMs are capable of capturing highly non-linear patterns between the response and predictors, as was expected and evident for two (slope and aspect) out of five environmental traits investigated here. Our global model included slope and aspect modelled with the thin plate regression splines, while solar radiation, elevation and distance to foraging hotspots were treated as linear (on the logit scale). All continuous predictors were uncorrelated, and scaled to help convergence. No collinearity was detected. GAM fitting was performed using *mgcv* library [27] in R (R Core Team 2015). 32 models were fitted in total with the *MuMIn* library [28], including the null model; models covered all possible combinations of the five predictors. Only the top four high-ranking models received substantial support with cumulative AIC weight of 0.988 (S2 Table). Relationships estimated from the four top-supported models were very similar (S2 Fig), thus model-averaging was unnecessary. We therefore present estimates and relationships from the top-supported model [29]. We also assessed the importance of predictors using the relative variable importance (RVI) concept, where RVIs are summed weights of models in which a given predictor occurs [30].

### Factors associated with the size of existing colonies

To model environmental variables asssociated with a number of nests in a colony patch, a Conditional Inference Tree (CIT) was used. CIT is a non-parametric class of regression trees, examining the relationship between multiple explanatory variables and a single response variable using a recursive binary-partitioning process. Model outputs produce an ‘inverted tree’, in which the root at the top contains all observations, which is divided into two branches at the node. The aim of splitting the data at each step is to establish groups that had a between-variation as large, and a within-variation as small, as possible. The node provides information about the explanatory variable name and its significance. Branches are further split into two subsequent nodes and so on [31]. CIT uses a machine learning algorithm to determine when splitting is no longer valid using a statistically-determined stopping criterion and an a priori *p* value. This is a non-parametric class of regression tree, robust to typical regression problems such as over-fitting, collinearity, and bias with regard to the types of explanatory variables used [32]. We performed CIT analysis with five quantitative (distance from the foraging hotspots, elevation, slope, aspect, May solar radiation [mean values per colony polygon]), and one factorial (rock type) predictors (the rock type was not dependent from the rock size). This analysis was performed using *partykit* library [32] in R (R Core Team 2015).

## Results

### Colony distribution and size

**Fig 4:**
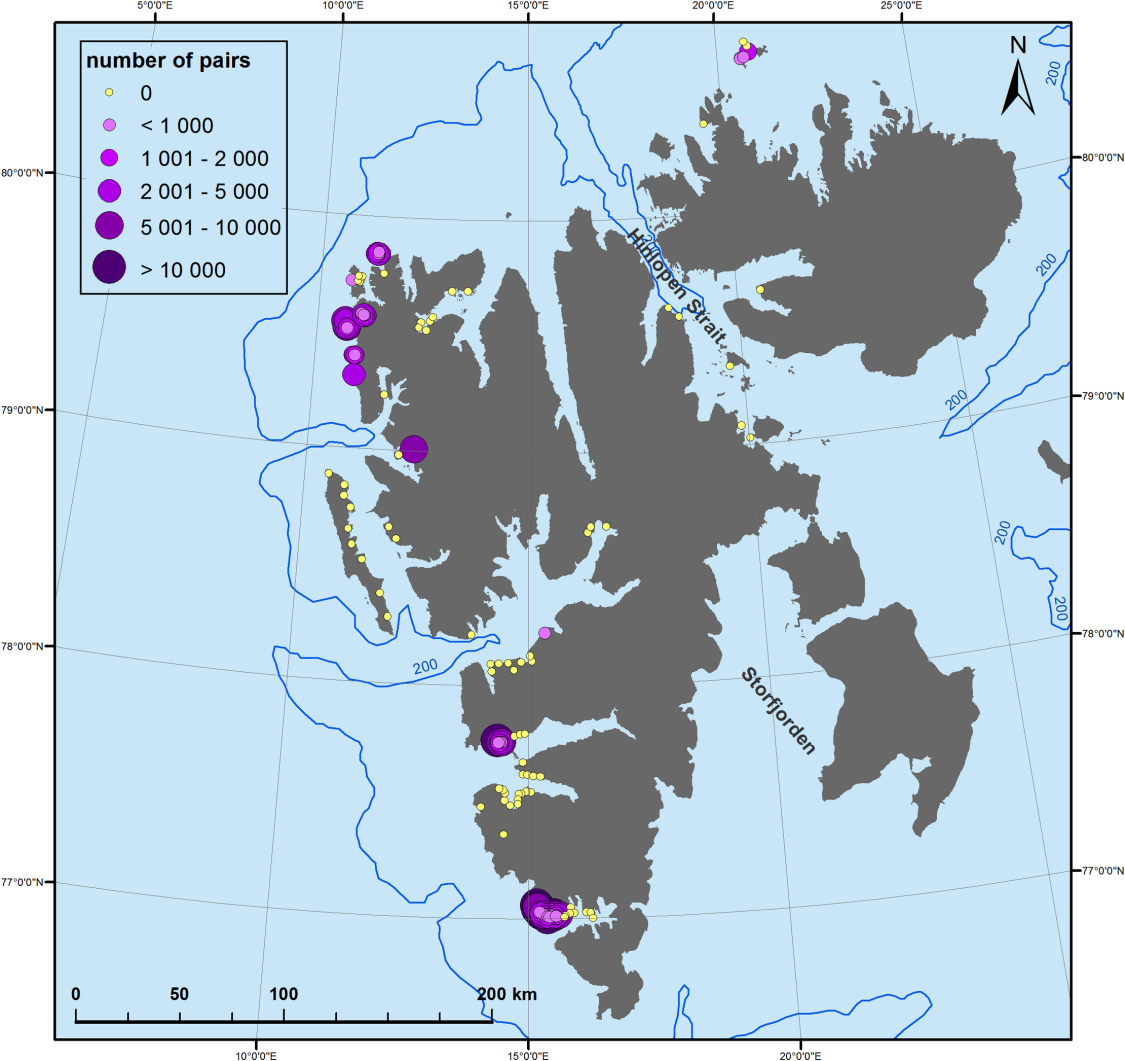
Little auk colony distribution in Spitsbergen. A size of the dot corresponds to a colony size (number of breeding pairs). Background (bathymetry and contour map): NOAA Satellite Maps.

The little auk colonies in W and NW Spitsbergen are concentrated mainly in three areas–Hornsund (S Spitsbergen), Bellsund (central Spitsbergen) and Magdanefjorden region (NE Spitsbergen) (Fig 4). The 143 visited colonies covered a total area of 0.415 km^2^, and accounted for an estimated number of 728 529 (5–95% CI 479 312–986 352) nests, with estimated nest density between 0.2 and 1.99 nest/m^2^, depending on a site (Table 3). An average colony patch covered 2 900 (SD = 7 859 m^2^), and hosted 5 094 nests on average (Table 3).

### Factors associated with colony presence/absence

**Fig 5:**
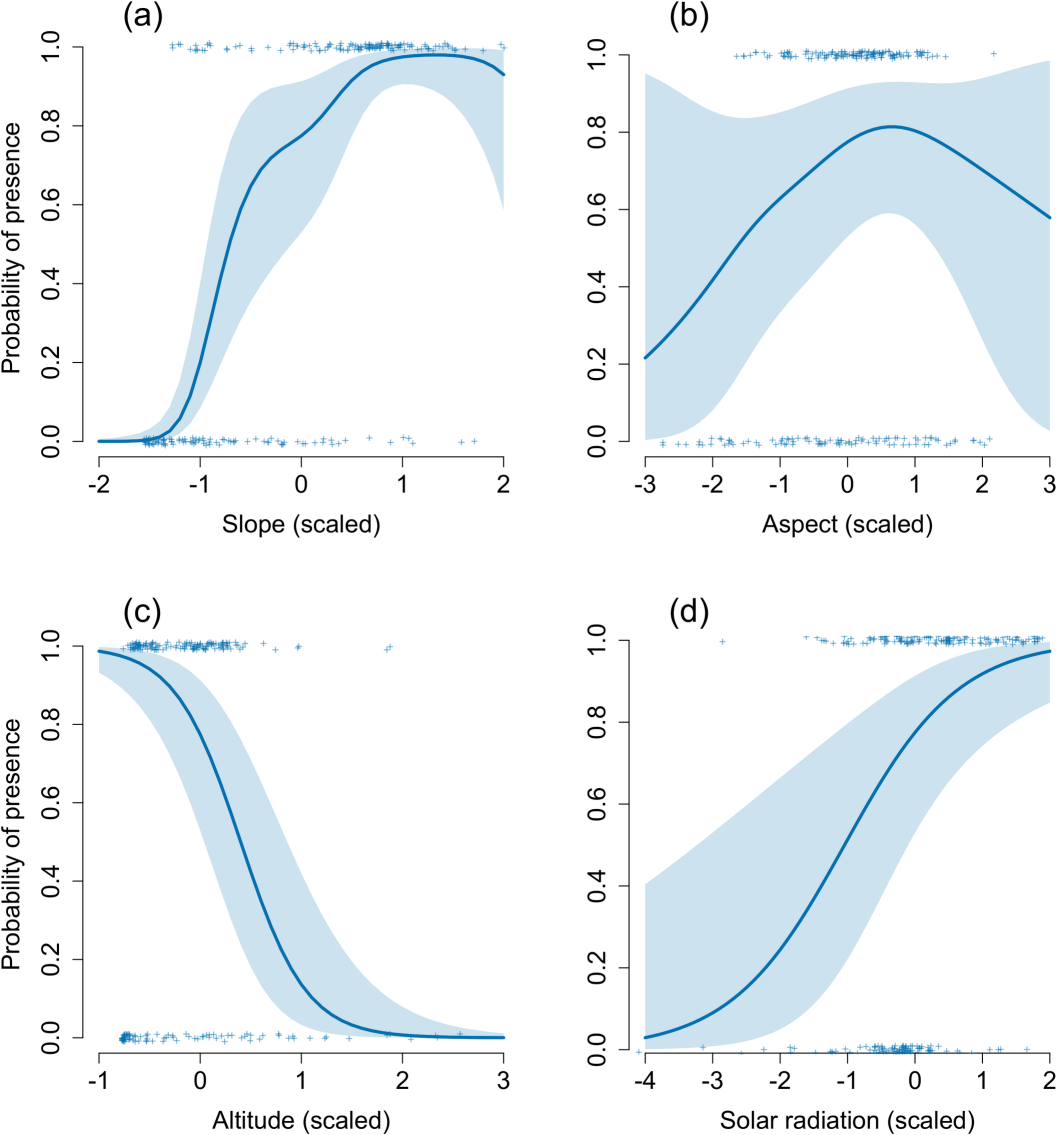
**Probability of colony occurrence in relation to slope angle (A), aspect (B), altitude (C) and solar radiation (D).** A and B are estimated smoothers, C and D–linear relationships on the logit scale. Bold line–estimated relationships, dashed lines–their 95% confidence intervals, crosses–data (0 –polygons, 1 –colonies).

Among 32 models fitted to the data, the top four models clearly outcompeted the remaining ones, with 99% of cumulative AIC weights (S2 Table). Estimated relationships were very similar (S2 Fig). The most important predictors were slope, elevation and solar radiation (RVI = 0.99 for all three), while aspect and distance to foraging hotspots were less important (RVI of 0.64 and 0.48, respectively). The top-supported GAM model explained 64% of the variance in the data. Birds showed a clear preference towards the steeper available slopes and avoidance of flat areas (spectrum of available slopes: 0–54°, average slope within the colonies is 28°). The probability of colony occurrence was significantly associated with elevation, with most true colonies located at low elevations. Solar radiation was also found to affect the probability of colony occurrence positively, with higher probabilities at locations with higher solar radiation. The effect of aspect was not significant due to broad confidence intervals around the estimate. However, the highest average probability of occurrence was estimated for intermediate values of aspect, that is, at southern directions (southern aspect falls within 157.5°–202.5°) (Fig 5 and Table 4).

**Table 4:** Parameter estimates of the best-supported GAM model explaining variation in colony presence/absence. Effective degrees of freedom (*edf*) is a measure of wiggliness of a smoother (a linear relationship on the logit scale has *edf* of 1).

**Effect**	**Estimate**	**SE**	**Z**	**p**
Intercept	0.409	0.275	1.490	0.136
Altitude	-3.083	0.595	-5.185	<0.001
Solar radiation*	1.183	0.376	3.148	<0.001
**Smoothers**	***edf***	**Ref df**	**χ**^**2**^	**p**
Slope	4.528	5.517	57.751	<0.001
Aspect	2.157	2.764	4.303	0.334

*—solar radiation was included as a sum for May-August.

### Factors associated with colony size

**Fig 6:**
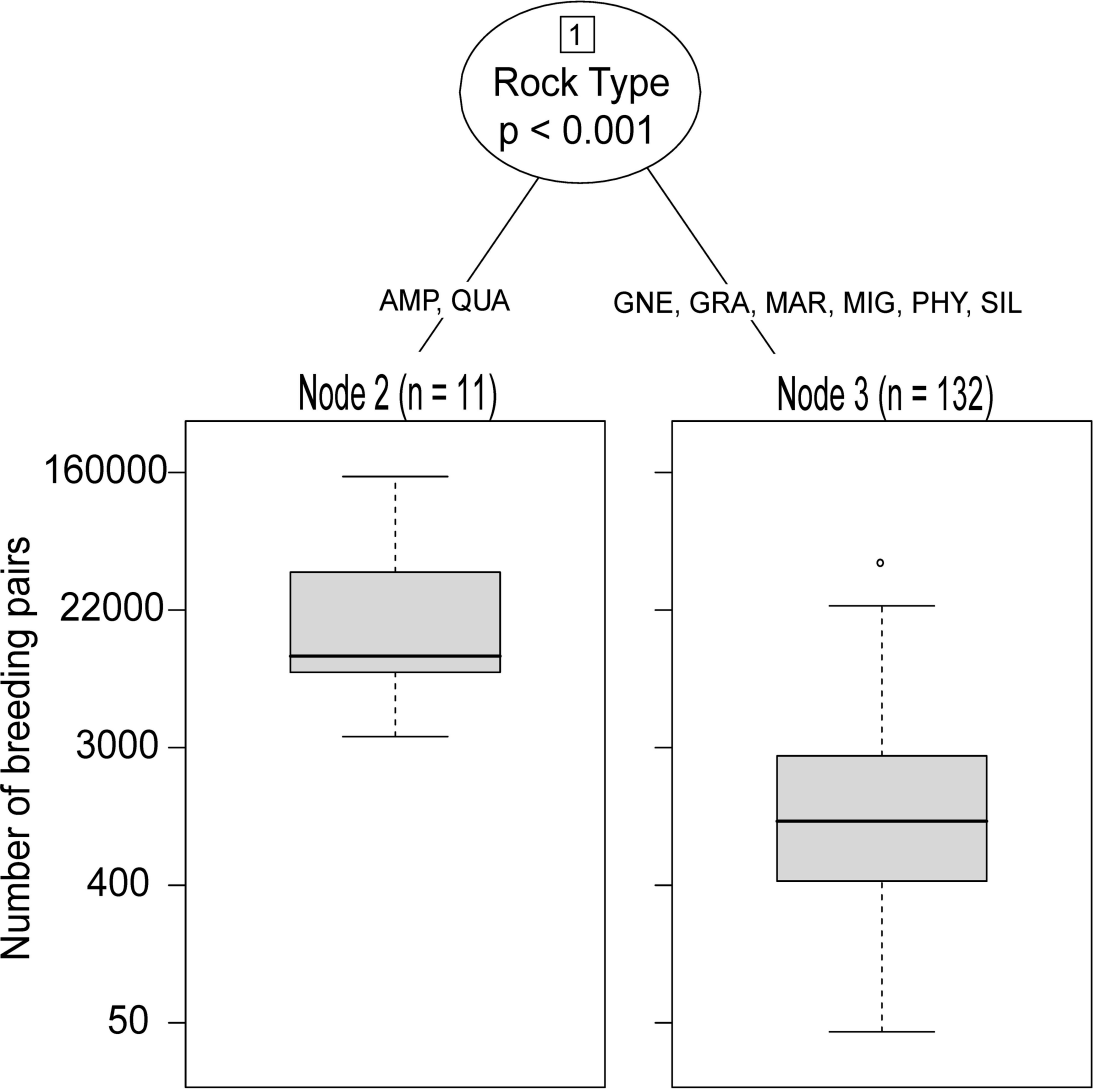
Conditional Inference Tree characterizing factors related to the size of the little auk colonies in W and NW coast of Spitsbergen (number of pairs) based on quantitative (altitude, slope, aspect, average solar insolation in May, distance to the nearest foraging hotspot [mean values per polygon]) and quantitative (type of rocks) predictors. Encircled variable is significantly (p < 0.001) related to the response variable (colony size). The p value shown in an encircled node represents the test of independence between the variable (type of rocks) and the response variable (colony size). N in terminal nodes indicates the number of colonies corresponding to specific type of rocks. Histograms in terminal nodes (nodes 2, 3) depict the size of colonies in particular groups differing in type of rocks. Boxplots show the median (band inside the box), the first (25%) and third (75%) quartile (box), the lowest and the highest values within 1.5 interquartile range (whiskers) and outliers (circles). Rock type codes: AMP–amphibolite, QUA- quarzite, GNE–gneiss, GRA–granite, MAR—marble, MIG–migmatite, PHY–phyllite, SIL–silt.

A Conditional Inference Tree characterizing little auk colony size (estimated number of breeding pairs) showed that among the studied quantitative (altitude, slope, aspect, average solar radiation in May, distance to the nearest foraging hotspot [mean values per polygon]) and qualitative (type of rock) predictors, only one variable: rock type, characterized significantly (p < 0.001) colony size (Fig 6). Colonies located in areas with prevalence of amphibolite and (AMP) and quartzite (QUA) were larger (Node 2; mean = 17 466, 11 colonies) than those located in areas with prevalence of other types of rocks (Node 3; mean = 1 134 breeding pairs, 132 colonies).

## Discussion

Our study confirms the importance of the W and NW coast of Spitsbergen as the little auk breeding grounds, with the greatest breeding aggregations concentrated in three regions: Hornsund (WS Spitsbergen), Bellsund (central Spitsbergen) and Magdanefjorden (NW Spitsbergen). This result is not suprising as all these areas have been recognized as breeding hotspots for the species (Norwegian Polar Institute, www.svalbardkartet.npolar.no). Nevertheless, we found differences when comparing the current distribution of colonies with the distribution reported historically [33]. At 14 sites where little auks have been reported to breed, we did not find evidence of their current presence. As there are no other seabird species exhibiting the same nesting preferences within the breeding range of little auks (burrows on mountain slopes), nest site competition is an unlikely factor driving the presence/absence of little auks. This discrepancy may instead be attributed to altering habitat properties, such as plant growth or rock erosion, limiting access to the nest burrows [34]. Plant overgrowth of slopes has been considered a likely reason driving the breeding site extinction in two Pacific alcids in Alaska [35,36]. However, as the discrepancy only concerned small colonies (<100 pairs) any conclusions about demography in those locations would be speculative, with small colonies typically prone to extinction solely by chance, without any observable changes in the environment [37,38].

Estimation of the little auk population size, regardless of geographical scale, is challenging due to nesting in deep rock crevices, often in inaccessible areas. Our method of combining documented nest densities with the measurements of rock sizes from the colonies is not free from caveats, stemming mostly from necessary simplifications, primarily because densities of nests may vary within any colony patch (every colony has its own confidence intervals, however it was not included in the analysis), depending on the local topography, rock sizes and other factors. However, even with these limitations, it is the first estimate relying on available empirical data on rock size, providing a formal measure of uncertainty of the estimate which makes it more reliable than any other documentation in this topic up to this date. We estimated the Svalbard population at 728 529 (5–95% CI 479 312–986 352) pairs, while previous estimates reported over 1 million pairs [13]. On the other hand, the whole Barents Sea region population (including Svalbard) has been estimated to 580 000 pairs by other authors [39]. With such an enormous range of estimates of the population size it is difficult to assess whether, firstly, any temporal changes have taken place, and, secondly, to predict future numbers. Nevertheless, considering our results, and the most recent estimates of the global population (37–40 million pairs, [8]), the Svalbard population of little auk accommodates approximately 1.9% (95% CI: 1.2%–2.7%) of the global population (Table 5). Even if the value *per se* may not seem large, particularly when compared with Greenland that hosts the core of the global population (~89%), it still represents an extremely important site for the species, which, in the Svalbard Archipelago is one of the most important components of the marine and terrestrial ecosystem. This is because the little auk is the most numerous seabird in the Svalbard area [17,40] and acts as an ecosystem engineer transporting marine-derived nutrients from sea to land, transforming terrestrial ecosystems and further affecting benthic coastal communities by a nutrient runoff from colony areas [10,41,42]. Given this, if recently proposed scenarios predicting noticeable decrease of the little auks in most Svalbard populations [12] occur, considerable ecosystem changes in various Svalbard regions are expected in future.

**Table 5:** Breeding population estimates for the little auks according to available literature and this study (non-additive).

Region	No. of breeding pairs	% of world population	Reference
***Svalbard***			
Svalbard (the whole archipelago)	>1 000 000	>3.7–4.0	[16]
Nordaust-Svalbard	1 190		[43]
Bjørnøya	10 000		[44]
Nordvest-Svalbard Nasjonalpark, Forlandet	1 500 000		[45]
Sør-Spitsbergen National Park (Hornsund)	20 100		[46]
Hornsund	200 000		[47]
Sør-Spitsbergen National Park (Hornsund)	886 000		[13,16]
Edgeøya	450		[48]
**Spitsbergen–this study, mean values**			
Bellsund	35 814		This study
Hornsund	591 892		This study
NW Spitsbergen	91 978		This study
Isfjorden (Bjørndalen) Kongsfjorden Sjuøyane	135 3 890 4 821		This study This study This study
the whole Spitsbergen	728 529	1.8–2.0	This study
***East Barents Sea***			
Whole Barents Sea region	>1 300 000	3.2–3.5	[33]
Whole Barents Sea region	580 000	1.4–1.5	[39]
Novaya Zemlya	11 000		[49]
Novaya Zemlya	50 000		[50]
Novaya Zemlya	30 000–50 000		[51–53]
Severnaya Zemlya	10 000–80 000		[54]
Franz Joseph Land	250 000		[55]
Franz Joseph Land	30 000–50 000		[17]
***Jan Mayen***	10 000–20 000		[50]
***Greenland***			
Whole Greenland	23 500 000–38 500 000	63.5–96.2	
Scoresby Sund (E Greenland)	>3 500 000		[15]
Thule (W Greenland)	20 000 000–33 000 000		[56,57]
Northumberland Island (Thule, W Greenland)	8 834 919		[14]
World population estimate	37 000 000–40 000 000	100	[8]

As we expected, the little auk colonies along the NW Spitsbergen coast showed a non-random distribution. The probability of colony occurrence was significantly associated with elevation, and was the highest at low and moderate altitudes and moderate at high altitudes. This was likely to be related to appropriate rock or substrate sizes being distributed mainly at low and moderate elevations [16,17,33]. Solar radiation was the next important factor associated with presence/absence of little auk breeding colonies on Svalbard. The colonies located in southern Spitsbergen were exposed to higher solar radiation values than those in NW Spitsbergen. This parameter may be easily translated to overall air temperature, particularly important for birds in spring, when it affects the timing of snow melt, which in turn affects the onset of the breeding season [58]. For example, median hatching date in Isfjorden (central W Spitsbergen) is usually >1 week earlier than in Hornsund (SW Spitsbergen [59]) due to an earlier nest chamber availability facilitated by snow melt. Slope aspect exhibited very broad confidence intervals (possibly due to a restricted range of aspect values in true colonies) and, although did not appear significant in the statistical sense, the highest probability of colony occurrence on average fell within W-SW aspects. Such a nest exposure was in line with expectations: favouring faster snow melting during spring, and acting in a similar way to that of solar radiation, may affect the probability of the little auk colony occurrence. The probability of colony occurrence was significantly associated with slope. Moderate slopes provide relatively stable ground, where rocks and stones sliding is limited. Moderate slopes are more likely to provide more nest chambers than steeper slopes or vertical cliffs. Moderate slopes also facilitate easier take-off, which may be particularly important for short-winged seabirds, like little auks, with an unfavourable ratio of wing area to body mass [17]. In this context, it was not surprising that the average value of slope recorded in the little auks colonies (28.4°) was similar to values reported for other alcids; the least and crested auklets (*Aethia pusilla*, *Aethia cristatella*) nesting in Pacific colonies [23° [60]].

Generally, seabird colony size and location depend to some degree on local food availability [61,62]. The waters off the W coast of Spitsbergen are considered to be optimal foraging grounds for little auks [63,64]. This is either because of the strong influence of cold Arctic currents running along the E coast of Spitsbergen and then along the W coast (Hornsund, Bellsund; S3 and S4 Figs) or a relatively close distance to the marginal ice zone (Magdalenefjorden; S5 Fig) offering a high availability of energy-rich zooplankton [65–67]. However, distance to foraging hotspots was not significantly related to either colony presence/absence probability or colony size, which is best explained by flexibility of little auks to forage in suboptimal or more distant feeding areas under unfavourable foraging conditions (e.g. [12,62]). In other words, it is possible, that despite considerable differences in location of the foraging hotspots in relation to the studied breeding colonies, these grounds are still within the foraging range of the little auk.

Our study revealed that local population size is associated with rock type. This finding may result from the patchy distribution of appropriate rock substrate [25]. Worth highlighting is, that the rock type is not dependent from the rock size. Regarding colonies found on sedimentary rocks in Isfjorden and Bellsund, a morphological difference can be observed. The average size of the rocks in Bellsund was approximately two times bigger compared to Isfjorden. In Bellsund the colonies were dense with >35 000 pairs of little auks, while the BjØrndalen (Isfjorden) colony was very small. This may be a result of mass wasting and mud slides that are more pronounced in this area than in other places, preventing the birds from establishing stable colonies that persist for many years. Mass wasting and rock erosion are known to be more pronounced in sedimentary rocks due to their porosity [25,68]. However, colonies in Bellsund comprise the third largest little auk concentration in Svalbard. The silts that build the colonies in Bellsund provide much more stable grounds than the sandstones in Isfjorden. Rocks in Bellsund are also larger and offer more burrow systems suitable for nesting than in Isfjorden. We conclude therefore, that the fast erosion of sandstones may limit the Isfjorden population.

The largest little auk colonies were found on metamorphic rocks (quartzite and amphibolite). The colonies in Hornsund contain mostly phyllites and quartzite, whereas in NW Spitsbergen they contain migmatite and granite (Bellsund and Isfjorden are built almost exclusively of sedimentary rocks). Our findings show that the birds form larger colonies on metamorphic rocks, probably due to the stability of the slopes and higher resistance to erosion (therefore, bigger rocks on the slope providing more nesting chambers) [68]. Little auks are gregarious and require colonial nesting for successful breeding, so they likely prefer locations with rock types where it is possible to establish large, dense colonies [17,34]. However, since different rock types are not evenly distributed along the W coast of Spitsbergen, the birds do not have the whole spectrum of rocks to choose from at every location. Therefore, conclusions about a preference towards a certain rock type should be treated with caution.

## Conclusions

With 728 529 (5–95% CI 479 312–986 352) breeding pairs in total, the W and the NW Spitsbergen populations of little auks represent the most important seabird aggregations in the Svalbard Archipelago. Spatial distribution of the colonies along Spitsbergen coast is not random but rather determined by elevation, solar radiation and slope. The size of the particular colonies is also associated with geomorphological factors such as rock type but not with the distance to foraging hotspots. Knowledge of breeding population distribution and size of this Arctic endemic species is crucial for current population status assessments and any future conservation management.

## Supporting information

S1 TableParameters estimated for the relationship between mean rock size and nest density using a Bayesian linear model ($\hat{D}$ = a + b * (log(*RS*)).(DOCX)

S2 Table Generalized additive linear models fitted to data to explain variation in little auk colony presence. ‘+’ indicates whether a given term is included in the model. Models are ranked according to AIC value (lower AIC = bigger support). All models contain an intercept (omitted in the table for clarity).
(DOCX)

S1 Fig Relationship between mean rock size and nest density according to the linear model applied in this work. Line–mean, grey area– 95% CrI, points–original observations. The bigger symbol denotes two observations.
(TIF)

S2 Fig Relationships between predictors and the probability of little auk colony occurrence in Spitsbergen according to top four models. Slope and aspect were modelled with smoothers, remaining relationships are linear on logit scale. The relationship from the top-supported model is shown with bold, remaining three top-supported models with thin curves. Note that not all predictors are present in all four models.
(TIF)

S3 Fig Detailed map with the colony distribution in Hornsund (65 colonies), southern Spitsbergen. Background (contour map): NOAA Satellite Maps.
(TIF)

S4 Fig Detailed map with the colony distribution in Bellsund (15 colonies), central Spitbsergen. Background (contour map): NOAA Satellite Maps.
(TIF)

S5 Fig Detailed map with the colony distribution in NW Spitsbergen (56 colonies). Background (contour map): NOAA Satellite Maps.
(TIF)

S6 Fig Mean size of the colonies (number of pairs) located in different types of rocks. The number of colonies within specific groups given above the columns. Yellow columns correspond to the second node in Conditional Inference Tree (Fig 6). Whiskers—standard deviation.
(TIF)

S1 Data Spreadsheet with colonies (presence: 1) and absence locations (absence: 2) and environmental factors mean values. (XLSX)

S2 Data GIS Shapefile with randomly selected polygons without colonies (absence). (ZIP)

S3 Data GIS Shapefile with colonies. (ZIP)

S4 Data GIS Shapefile with foraging hotspots centroids. (ZIP)
