# Supplementary material for: Determinants of the little auk (Alle alle) breeding colony location and size in W and NW coast of Spitsbergen
Source: PLoS One. 2019 Mar 6;14(3):e0212668. doi: 10.1371/journal.pone.0212668 (PMC6402645; doi:10.1371/journal.pone.0212668)
Supplement: S1 Table — (DOCX) [file pone.0212668.s001.docx]

S1 Table. Parameters estimated for the relationship between mean rock size and nest density using a Bayesian linear model (*D* = a + b * (log(size)).

| Parameter | Mean ± SD | 95% CrI | *R*-hat (upper CI) |
| --- | --- | --- | --- |
| Intercept (a) | -4.597 ± 2.117 | -8.762; -0.377 | 1.006 (1.024) |
| Slope (b) | 1.606 ± 0.611 | 0.412; 2.823 | 1.007 (1.026) |
