# Supplementary material for: Determinants of the little auk (Alle alle) breeding colony location and size in W and NW coast of Spitsbergen
Source: PLoS One. 2019 Mar 6;14(3):e0212668. doi: 10.1371/journal.pone.0212668 (PMC6402645; doi:10.1371/journal.pone.0212668)
Supplement: S2 Table — ‘+’ indicates whether a given term is included in the model. Models are ranked according to AIC value (lower AIC = bigger support). All models contain an intercept (omitted in the table for clarity). (DOCX) [file pone.0212668.s002.docx]

| Model | linear | | | smoothers | | df | AIC | Δ AIC | ω AIC |
| --- | --- | --- | --- | --- | --- | --- | --- | --- | --- |
|  | distance | elevation | solar | aspect | slope |  |  |  |  |
| **1** |  | **+** | **+** | **+** | **+** | **10** | **132.4** | **0.00** | **0.329** |
| **2** | **+** | **+** | **+** | **+** | **+** | **11** | **132.5** | **0.09** | **0.315** |
| **3** |  | **+** | **+** |  | **+** | **7** | **133.6** | **1.19** | **0.181** |
| **4** | **+** | **+** | **+** |  | **+** | **8** | **133.8** | **1.41** | **0.163** |
| 5 | + | + |  | + | + | 11 | 139.9 | 7.55 | 0.008 |
| 6 |  | + |  | + | + | 10 | 140.9 | 8.50 | 0.005 |
| 7 | + | + |  |  | + | 8 | 150.2 | 17.83 | 0.000 |
| 8 |  | + |  |  | + | 7 | 150.8 | 18.43 | 0.000 |
| 9 |  |  | + | + | + | 15 | 183.1 | 50.77 | 0.000 |
| 10 | + |  | + | + | + | 10 | 184.6 | 52.19 | 0.000 |
| 11 |  |  |  | + | + | 14 | 184.6 | 52.26 | 0.000 |
| 12 | + |  |  | + | + | 10 | 185.5 | 53.10 | 0.000 |
| 13 | + |  | + |  | + | 8 | 192.8 | 60.48 | 0.000 |
| 14 |  |  | + |  | + | 7 | 196.8 | 64.41 | 0.000 |
| 15 | + |  |  |  | + | 7 | 201.1 | 68.70 | 0.000 |
| 16 |  |  |  |  | + | 6 | 206.1 | 73.70 | 0.000 |
| 17 | + | + | + | + |  | 9 | 256.2 | 123.87 | 0.000 |
| 18 |  | + | + | + |  | 8 | 262.0 | 129.62 | 0.000 |
| 19 | + |  | + | + |  | 8 | 263.4 | 131.00 | 0.000 |
| 20 | + | + |  | + |  | 8 | 267.9 | 135.56 | 0.000 |
| 21 | + | + | + |  |  | 4 | 268.6 | 136.23 | 0.000 |
| 22 | + |  |  | + |  | 6 | 270.7 | 138.30 | 0.000 |
| 23 |  |  | + | + |  | 7 | 271.2 | 138.87 | 0.000 |
| 24 | + |  | + |  |  | 3 | 277.2 | 144.86 | 0.000 |
| 25 |  | + | + |  |  | 3 | 277.7 | 145.36 | 0.000 |
| 26 |  | + |  | + |  | 7 | 280.1 | 147.78 | 0.000 |
| 27 |  |  |  | + |  | 5 | 284.0 | 151.61 | 0.000 |
| 28 |  |  | + |  |  | 2 | 288.7 | 156.36 | 0.000 |
| 29 | + | + |  |  |  | 3 | 294.3 | 161.93 | 0.000 |
| 30 | + |  |  |  |  | 2 | 298.4 | 166.05 | 0.000 |
| 31 |  | + |  |  |  | 2 | 313.1 | 180.70 | 0.000 |
| 32 |  |  |  |  |  | 1 | 318.8 | 186.45 | 0.000 |
