# Supplementary figures and images for: Determinants of the little auk (Alle alle) breeding colony location and size in W and NW coast of Spitsbergen
Source: PLoS One. 2019 Mar 6;14(3):e0212668. doi: 10.1371/journal.pone.0212668 (PMC6402645; doi:10.1371/journal.pone.0212668)

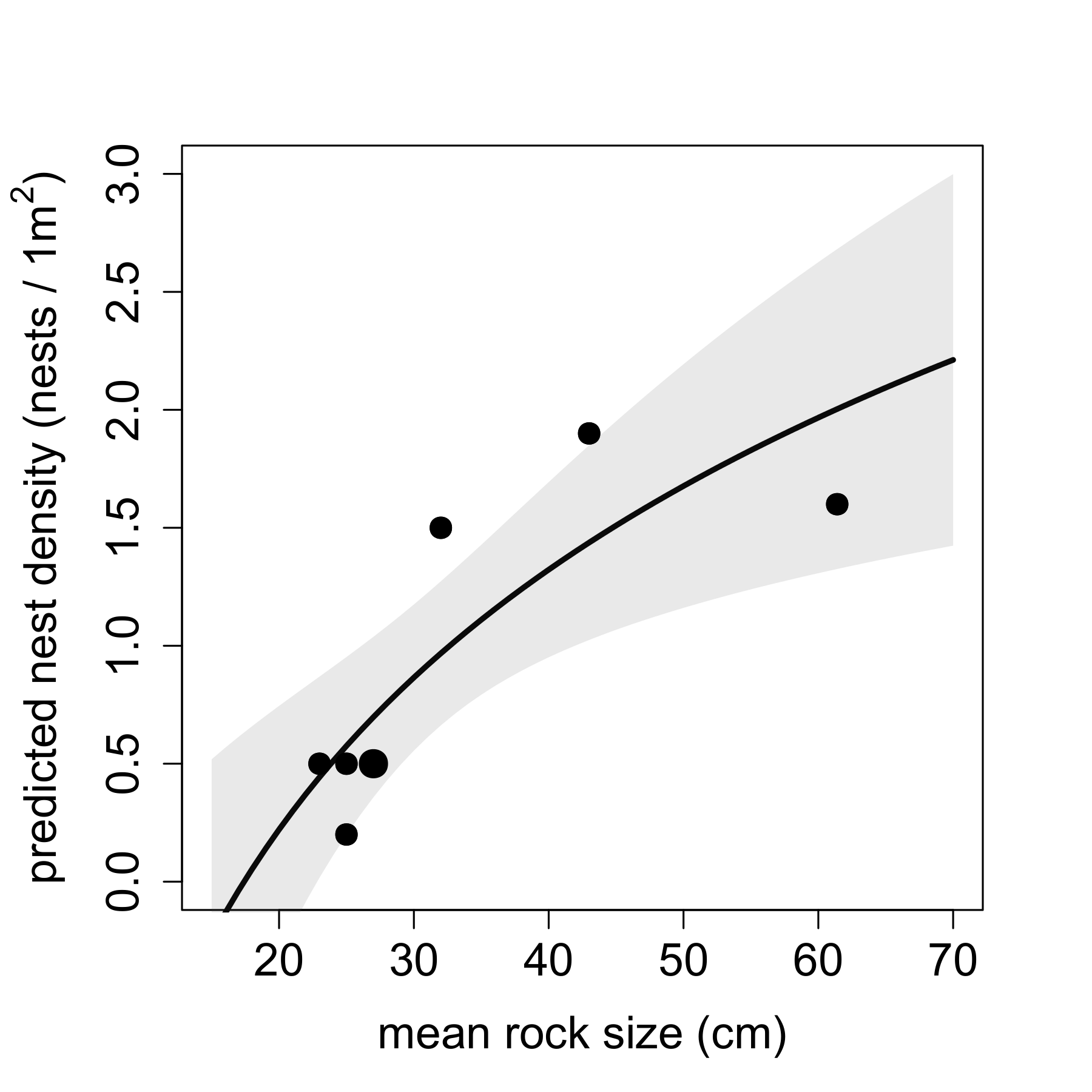

Supplement: S1 Fig — Line–mean, grey area– 95% CrI, points–original observations. The bigger symbol denotes two observations. (TIF) [file pone.0212668.s003.tif]

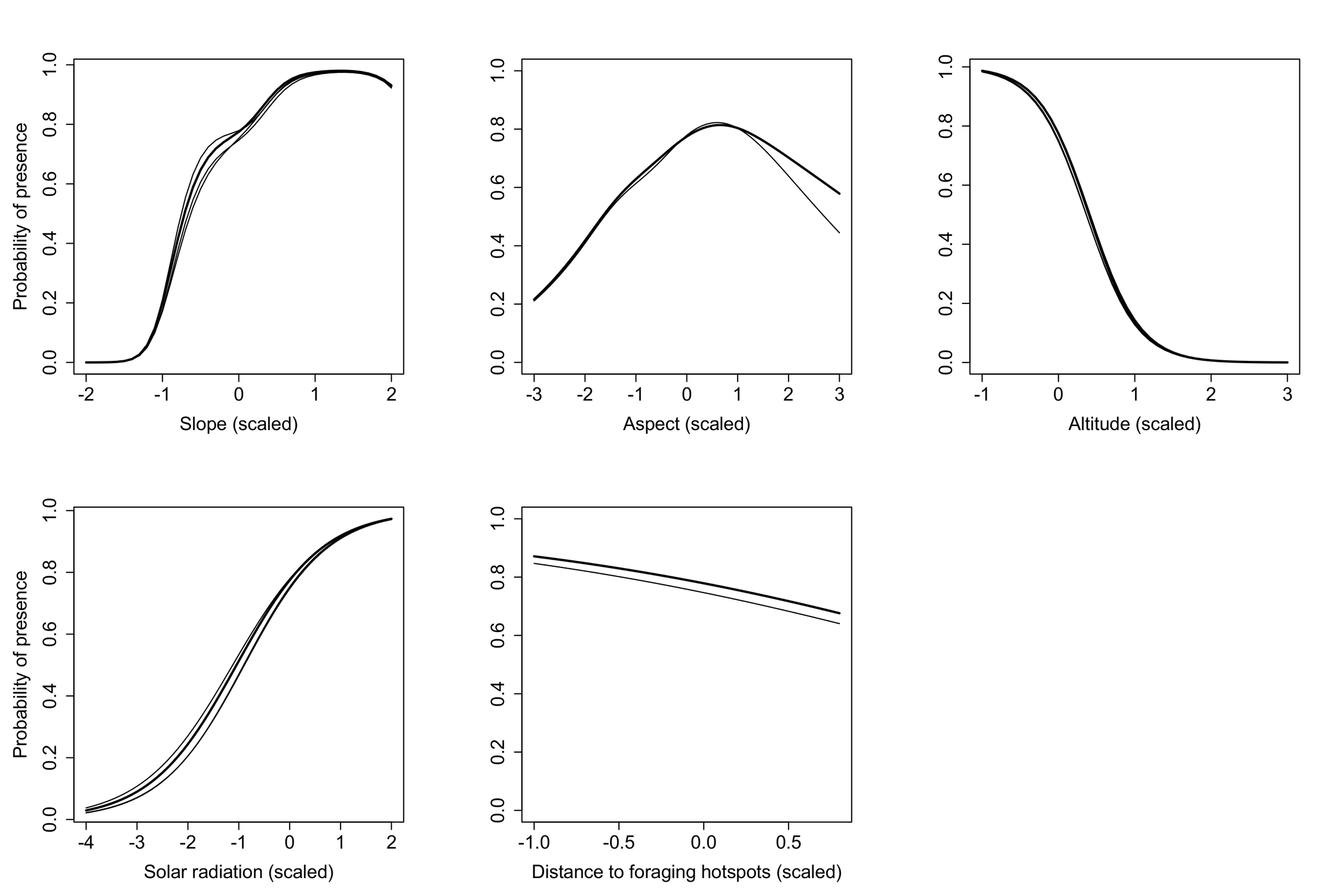

Supplement: S2 Fig — Slope and aspect were modelled with smoothers, remaining relationships are linear on logit scale. The relationship from the top-supported model is shown with bold, remaining three top-supported models with thin curves. Note that not all predictors are present in all four models. (TIF) [file pone.0212668.s004.tif]

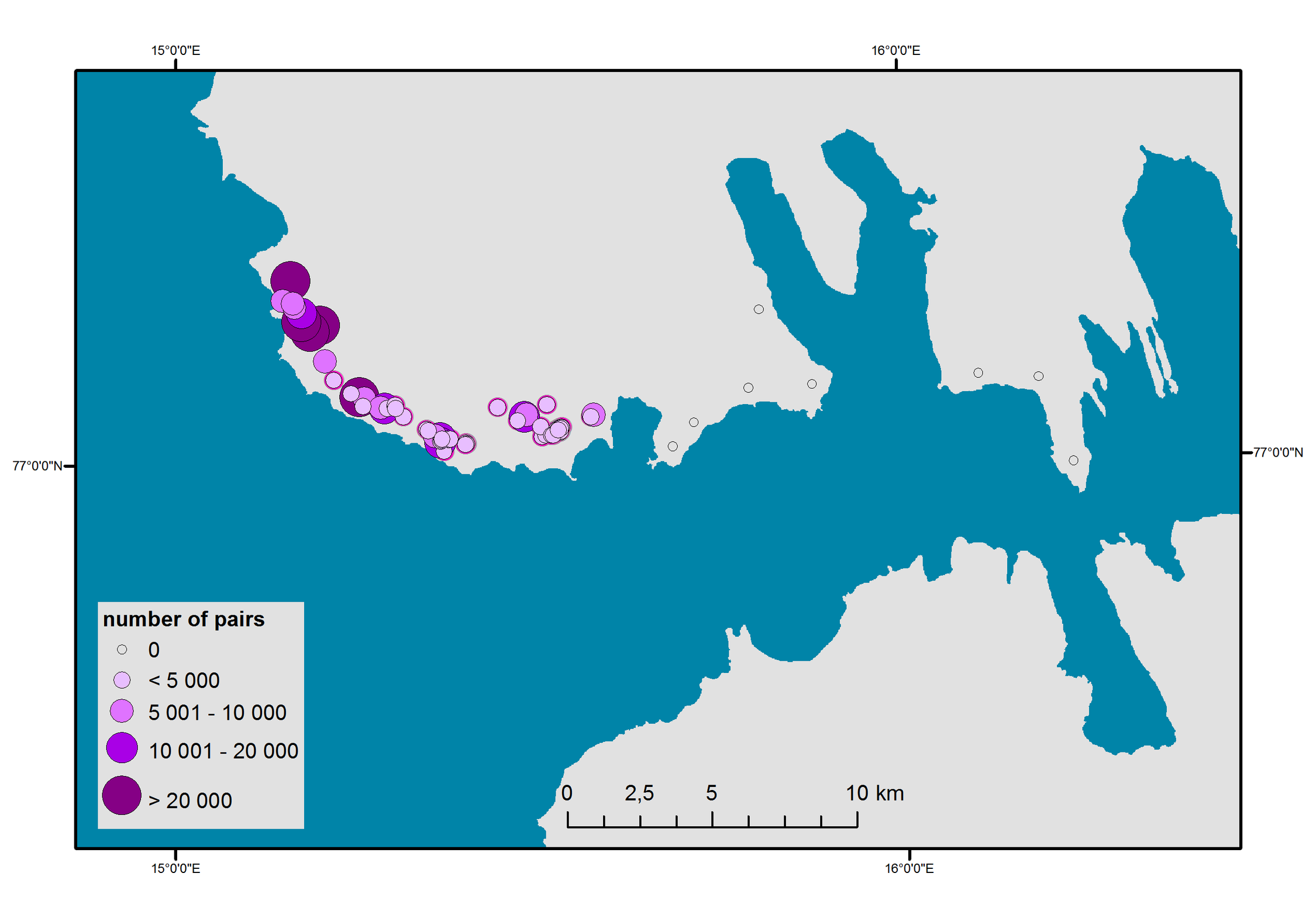

Supplement: S3 Fig — Background (contour map): NOAA Satellite Maps. (TIF) [file pone.0212668.s005.tif]

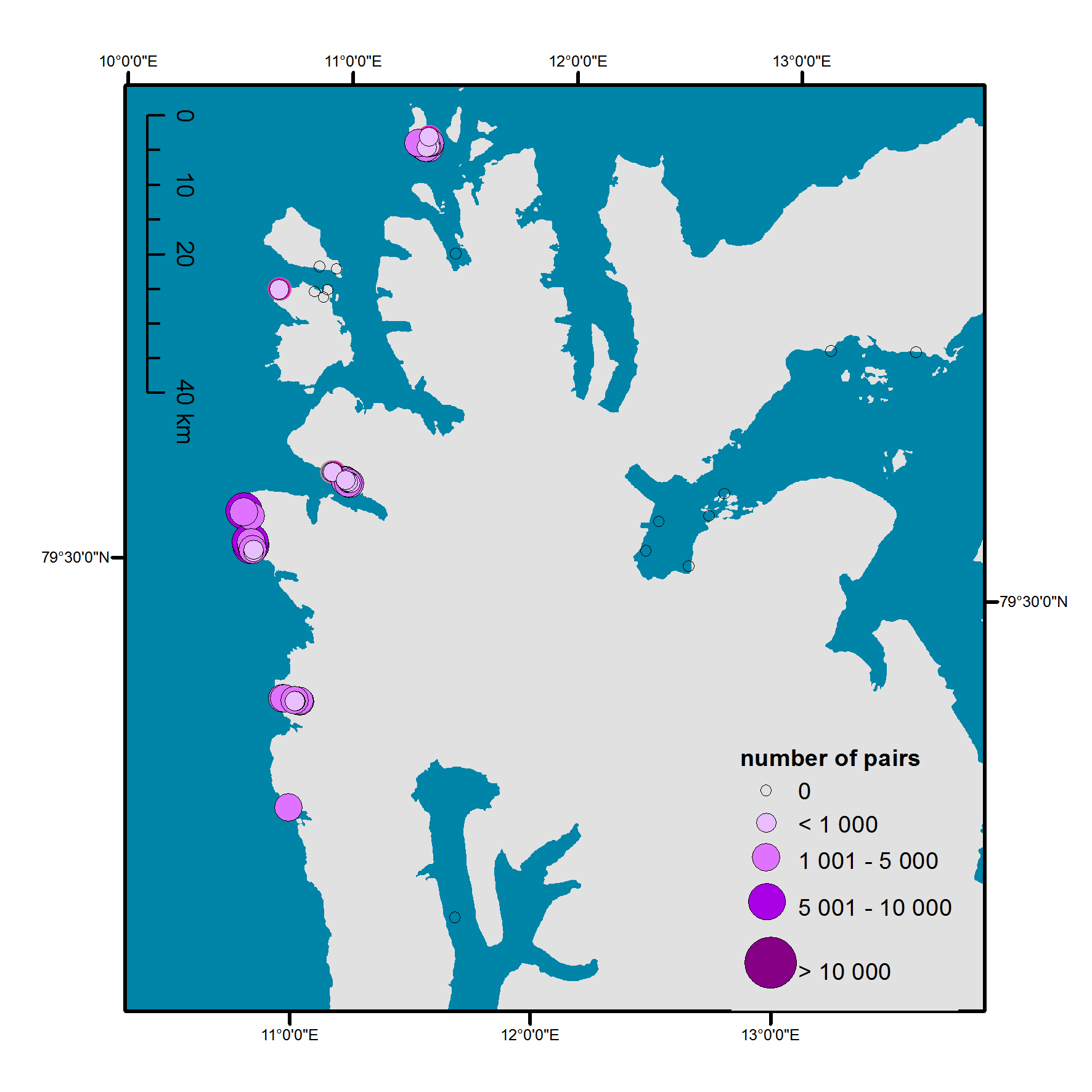

Supplement: S4 Fig — Background (contour map): NOAA Satellite Maps. (TIF) [file pone.0212668.s006.tif]

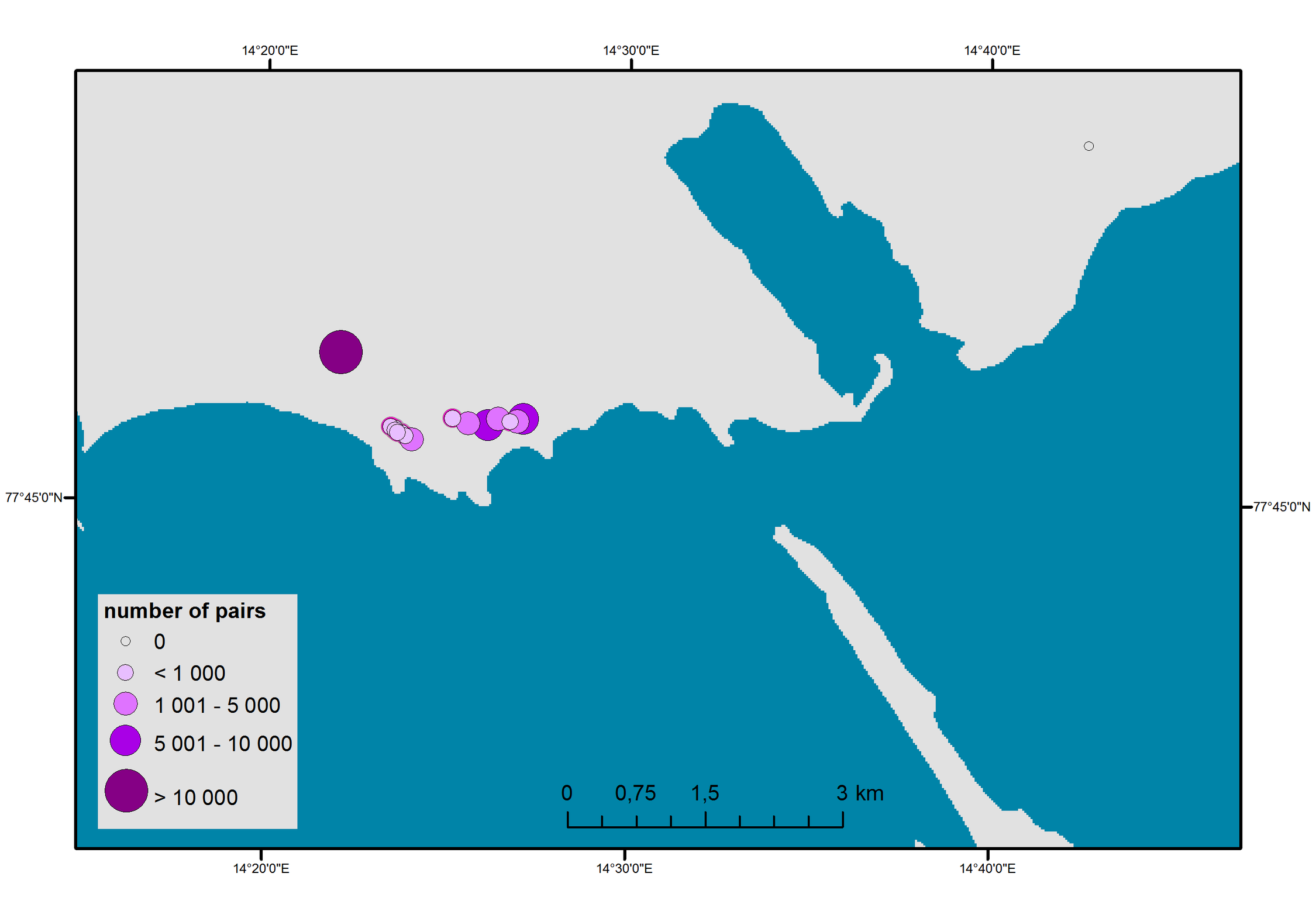

Supplement: S5 Fig — Background (contour map): NOAA Satellite Maps. (TIF) [file pone.0212668.s007.tif]

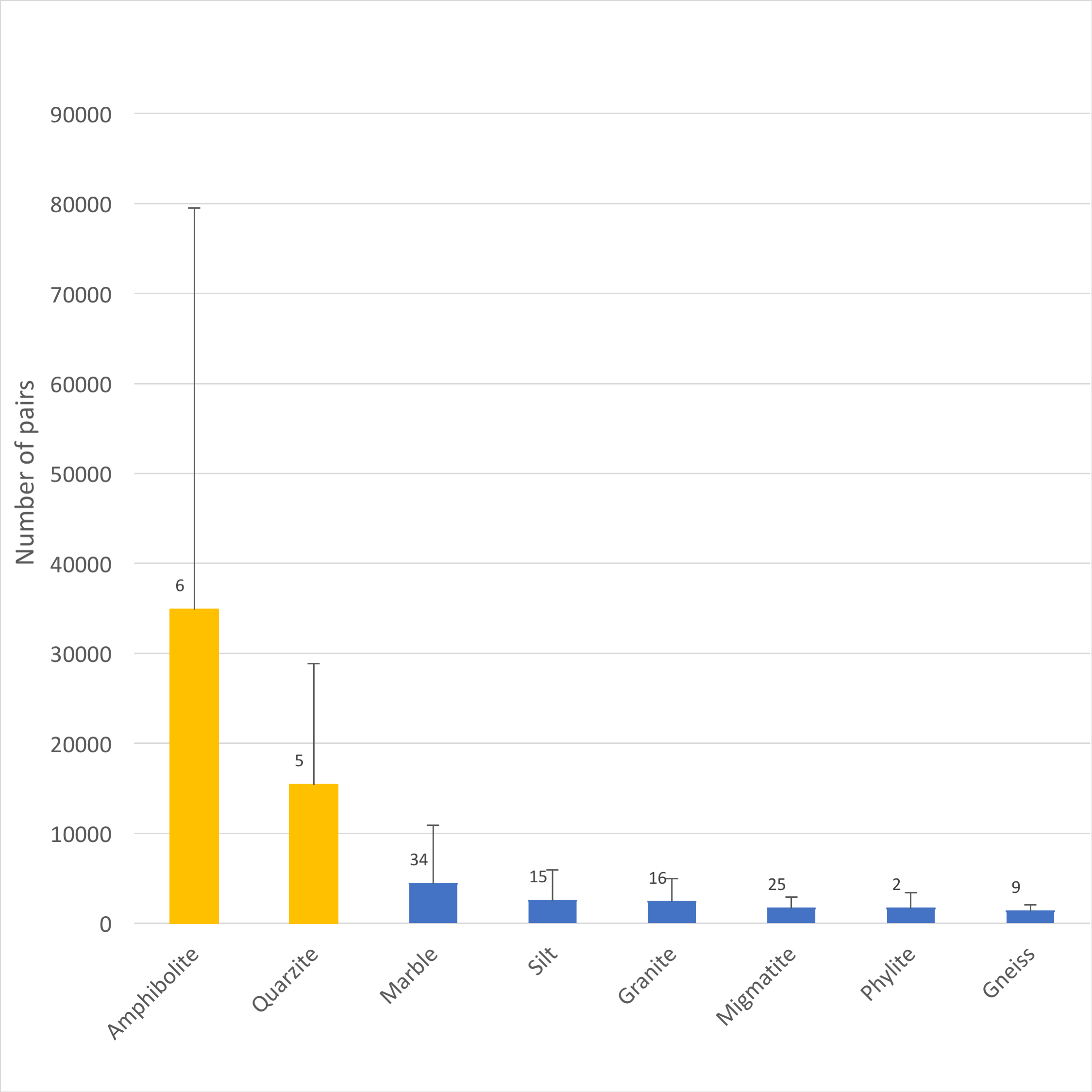

Supplement: S6 Fig — The number of colonies within specific groups given above the columns. Yellow columns correspond to the second node in Conditional Inference Tree (Fig 6). Whiskers—standard deviation. (TIF) [file pone.0212668.s008.tif]
